# Effects of Hypoxia on Nitric Oxide (NO) in Skin Gas and Exhaled Air

**Published:** 2006-09

**Authors:** Tetsuo Ohkuwa, Tatsuo Mizuno, Yuji Kato, Kazutoshi Nose, Hiroshi Itoh, Takao Tsuda

**Affiliations:** 1 *Department of Life and Materials Engineering, Nagoya Institute of Technology, Showa-ku, Nagoya, Japan;*; 2 *Pico Device Co., Ltd., Offices, Incubation Center, Nagoya Institute of Technology, Showa-ku, Nagoya, Japan*

**Keywords:** nitric oxide, hypoxia, skin gas, exhaled air

## Abstract

This study confirmed the effects of hypoxia on nitric oxide (NO) concentrations in skin gas and exhaled air. NO concentrations in skin gas and exhaled air were measured by a chemiluminescence analyzer. Arterial oxygen saturation (SpO_2_) of the right forefinger was determined using an oxygen saturation monitor. The M ± SEM of NO concentrations in skin gas at 20.93% (control), 15.1% and 14.8% oxygen concentrations were 23.7 ± 3.6, 32.3 ± 4.7 and 36.2 ± 5.2 ppb, respectively. M ± SEM of NO concentrations in exhaled air at 20.93% (control), 15.1%, and 14.8% were 25.0 ± 5.1, 35.01 ± 5.6 and 44.9 ± 7.2 ppb, respectively. There was no significant difference in NO concentration at the absolute value of skin gas and exhaled air between normoxia and hypoxia. But significant increase was found at relative changes in skin gas at 15.1% (*p*<0.01) and 14.8% (*p*<0.01) oxygen content compared with control. Significant increase was also found at relative changes in exhaled air at 15.1% (*p*<0.01) and 14.8% (*p*<0.01) oxygen content compared with control. In conclusion, we confirmed that exposure to hypoxia elicits an increase in NO concentrations at relative changes of skin gas and exhaled air compared to normoxia.

## INTRODUCTION

It has been reported that nitric oxide (NO) in exhaled air reflects the dynamics of NO production and consumption in the lungs (1, 2). NO is synthesized in various types of cells, such as endothelial, neutrophils, epithelial and autonomic nerves. NO originates close to the pulmonary vascular endothelium (3), the terminal and respiratory bronchioles (4), or the nasal epithelium (5), and it plays several important roles in the prevention of platelet aggregation, neurotransmission, the regulation of vascular tone, and blood pressure (6, 7). Others have demonstrated that the major site of NO output in the exhaled air of humans is the nasal airways (5, 8, 9). The concentration of NO in exhaled breath depends on several factors, such as inflammatory diseases (8, 10), physical exercise (4, 11), exercise training (12), smoking (5, 13), breath-holding (4, 9), hypoxia (2, 14), and hyperventilation (4).

Few reports have addressed the effects of hypoxia on exhaled NO concentrations. Beall *et al*. (2) demonstrated that the exhalation of nitric oxide by chronically hypoxic highlanders is greater than lowlanders. Oriveira *et al*. (15) reported that sheep skin NO and its metabolites are increased in burned wounds compared to non-burned skin. We previously detected methane, ethylene, ethane, and ammonia gases emanating from human skin (16, 17). No reports have referenced the effect of hypoxia on NO concentration in skin gas. The aim of the present study is to investigate the effects of hypoxia on NO concentrations in skin gas and exhaled air.

## METHODS

### Subjects

Participants for this experiment were fourteen healthy, nonsmoking, moderately trained male swimmers who swam at least 4~6 times a week. The study’s purpose, protocol, and possible risks were fully explained before each subject signed an informed consent agreement. The mean and standard error of mean (M ± SEM) of age, height, weight and mean body mass index (BMI) of the participants were 21.4 ± 0.97 years, 171.1 ± 1.54 cm, 67.3 ± 1.82 kg, and 22.9 ± 0.64 kg/m^2^, respectively.

### Experimental protocol

**Figure 1:**
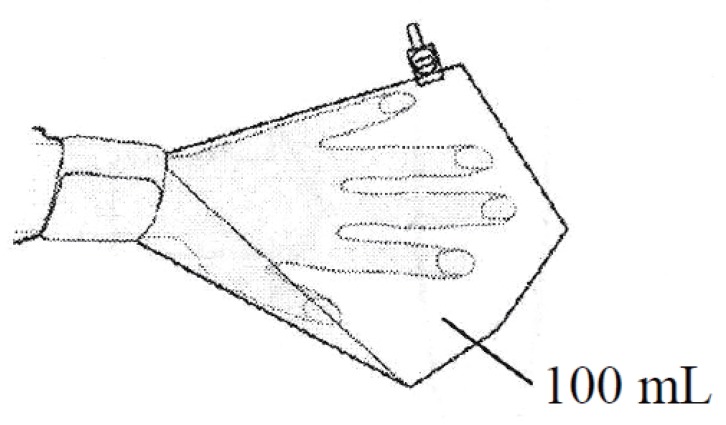
Diagrammatic representation of a skin gas collector.

Subjects sat comfortably and breathed normally wearing a nose clip for ten min in normoxic (20.93%) and hypoxic conditions (15.4, 15.1, and 14.8% oxygen concentrations for 20 min). Exhaled air was collected in a gas sampling bag (Tedlar bag; GL Science, Tokyo, Japan) that had been washed with pure nitrogen. Subjects breathed normally for 30 sec and were then asked to take one more normal breath. While the exhaled air was being collected, skin gas was also collected in a gas sampling bag (Tedlar bag) from the left hand. Skin gas was collected by the modified methods described in our previous study (16). The left hand was washed with running tap and distilled water, wiped with paper, and inserted into the sampling bag. Next, the sampling bag was washed twice with pure nitrogen gas. 100 ml of pure nitrogen gas was injected into the sampling bag, as shown in Fig. 1. Skin gas was collected by covering the left hand for three minutes with skin gas sampling bags made of a polyvinyl fluoride sheet (GL Science, Tokyo, Japan). The left wrist was fixed with a flexible sealing film and a band to prevent skin gas leakage through the space between the bag and skin.

To calculate the hand’s total surface area, the length of each finger and its circumference at the center point was measured. The surface areas of the palm and back of the hand were calculated from the length of one side, the other side, and the thickness.

An hypoxic environment was maintained using a normobaric hypoxic chamber. The hypoxic control system consisted of an oxygen control unit (YHS-CO5 B, YKS, Nara, Japan) and an air compressor (SLP-22 CO, YKS).

NO concentrations in the skin gas and exhaled air were measured by a chemiluminescence analyzer (Hamamatsu Photonics, Hamamatsu, Japan), using continuous samplings from a mixing chamber at a rate of 260 ml/min. The analyzer’s detection limit was 1 ppb for NO, with a 90% response time of about 1 sec. The analyzer was calibrated before each measurement using pure nitrogen and five certified NO concentrated gases (159, 79.8, 39.9, 19.9, and 9.98 ppb). Arterial oxygen saturation (SpO_2_) of the right forefinger was determined using an oxygen saturation monitor (Pulsox-2; Konika Minolta, Osaka, Japan).

### Statistical analysis

Values were expressed as mean and standard errors of the mean (M ± SEM) of the duplicate measurements of each sample. Data were analyzed using a one-way analysis of variance with repeated measures with *p*<0.05 determined to be statistically significant. When differences were obtained, post hoc analyses were performed using Sheffe’s F. Statistical analysis was calculated with Stat View (Ver. 5.0, Avacuus Concepts Inc).

## RESULTS

**Figure 2:**
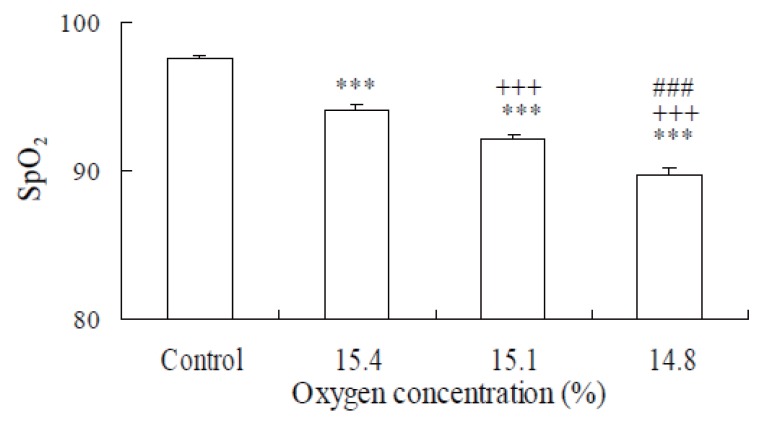
Effects of hypoxia on SpO_2_ Values are means ± SEM. ***p<0.001, significant difference compared with control. ^+++^*p*<0.001, significant difference compared with 15.4% oxygen concentration. ^###^*p*<0.001, significant difference compared with 15.1% oxygen concentration.

Figure 2 represents the changes in the SpO_2_ levels in the inhalation of 20.93% oxygen (normoxia), 15.4%, 15.1% and 14.8% oxygen. SpO_2_ levels with oxygen inhalation of 15.4%, 15.1% and 14.8% were significantly lower than normoxia (*p*<0.05 and *p*<0.001).

**Figure 3:**
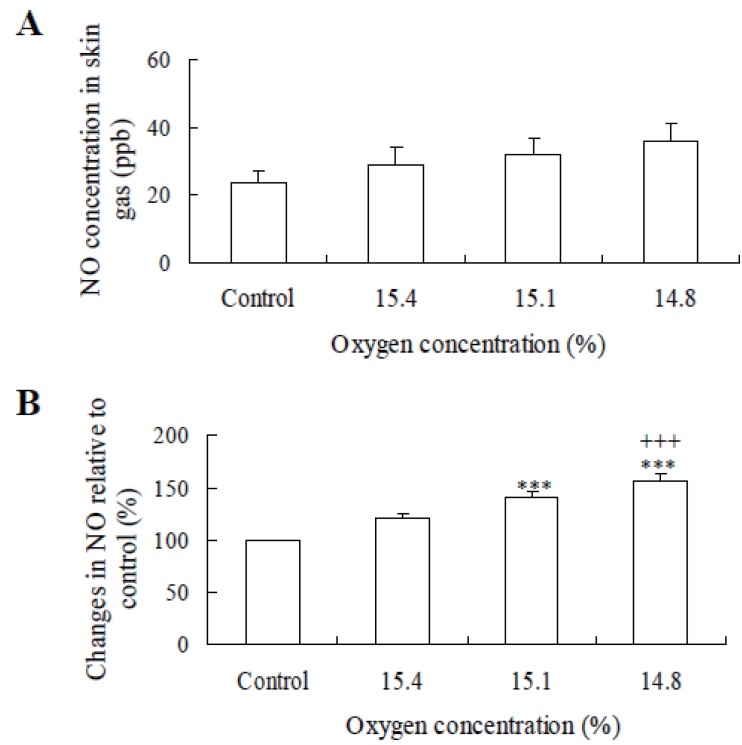
(A) Effects of hypoxia on NO concentration in skin gas (A). (B) represents % increase of NO in skin gas (%). Values are means ± SEM. ***p<0.001 significant difference compared with control. ^+++^*p*<0.001 significant difference compared with 15.4 oxygen concentration.

**Figure 4:**
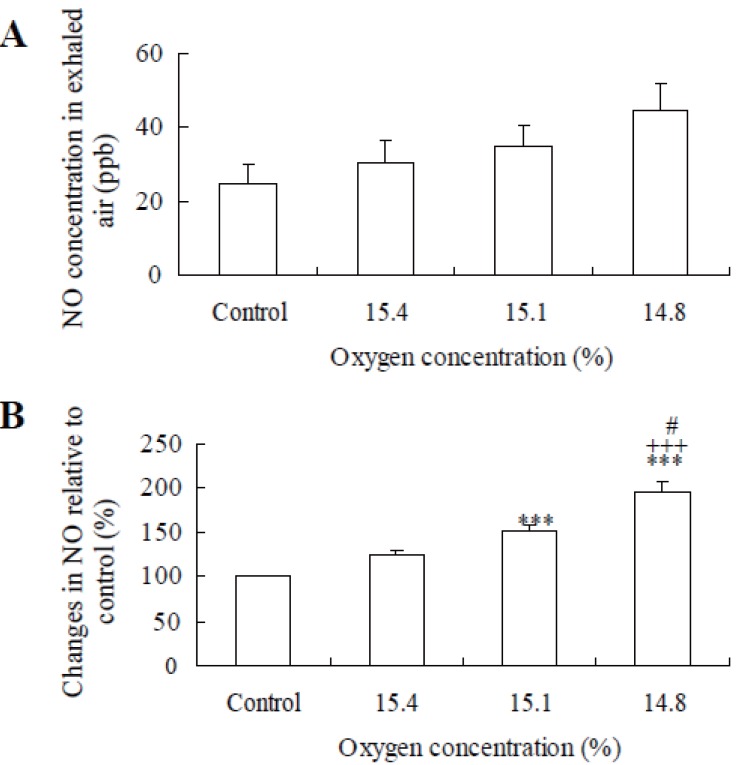
(**A**) Effects of hypoxia on NO concentration in skin gas. (**B**) represents % increase of NO in expired air (%). Values are means ± SEM. ***p<0.001 significant difference compared with control. ^+++^*p*<0.001 significant difference compared with 15.4% oxygen concentration. ^#^*p*<0.05 significant difference compared with 15.1% oxygen concentration.

Figure 3 shows the effects of hypoxia on NO concentration in skin gas whose skin gas NO level tended to increase with a decrease of the oxygen concentration of inhalation, but it was not significant (Fig. 3A). Figure 3B represents the percentage changes of the value at normoxia. Under hypoxia of 15.1% and 14.8%, NO concentrations in skin gas were significantly higher than normoxia (Fig. 3B) (*p*<0.05 and *p*<0.001). The exhaled air NO level tended to increase with a decrease of the inhalation of oxygen concentration, but it was not significant (Fig. 4A). Under hypoxia of 15.1% and 14.8%, NO concentrations in expired air were significantly higher than normoxia (Fig. 4B) (*p*<0.05 and *p*<0.01). Oxygen content was not found to correlate significantly with NO concentrations in skin gas (r=–0.281) and in exhaled air (r=–0.195). The M ± SEM of the total hand area in subjects (n=8) was 752.8 ± 17.80 cm^2^. The M ± SEM of NO concentration was 34.41 ± 9.60ppb. No significant correlation was found between NO concentration and the hand’s surface area(r=0.024, n=8). NO concentration/surface area of the hand (ppb/cm^2^) related to NO concentration (r=0.996, *p*<0.01). These results suggest that NO concentration is independent on the surface area of the hand.

## DISCUSSIONS

The present study indicates that SpO_2_ was significantly lower in hypoxia compared with normoxia, suggesting that moderate exposure to hypoxia induced SpO_2_ reduction, which basically agrees with previous reports (18, 19).

There was no significant difference in NO concentration at the absolute value of skin gas and exhaled air between normoxia and hypoxia (Figs. 3A and 4A). But significant increase was found at relative changes in skin gas at 15.1% (*p*<0.01) and 14.8% (*p*<0.01) oxygen content compared with control (Fig. 3B). Significant increase was also found at relative changes in exhaled air at 15.1% (*p*<0.01) and 14.8% (*p*<0.01) oxygen content compared with control (Fig. 4B).

This study clarified that hypoxia increases NO concentrations in exhaled air and skin gas. Our results are consistent with Hample *et al*. (14), who showed that acute hypoxia induced NO production increases in cultured bovine pulmonary arterial endothelial cells. Beall *et al*. (2) also reported that exhaled NO concentrations were higher in the chronically hypoxic populations of Tibetans living at 4,200 m than in lowlanders. Strijdom *et al*. (20) demonstrated that hypoxia activates NO production increases in cardiac microvessel endothelial cells. Justices *et al*. (21) found that hypoxia increased NO production, endothelial NO synthase protein, and endothelial NO synthase mRNA in epicardial arteries. Arnet *et al*. (22) also demonstrated that exposure to hypoxia induces increases in endothelial NO synthase mRNA and protein expression in bovine aortic endothelial cells. In contrast to the above reports of hypoxia-induced NO production increase, Whorton *et al*. (23) demonstrated that exposure to hypoxia caused a decrease in the NO production of bovine aortic endothelial cells. Hong *et al*. (24) also reported that hypoxia inhibited NO production in smooth muscle. On the other hand, Tsujino *et al*. (25) concluded that the inhalation of hypoxic gas did not cause any significant change in NO concentration in exhaled air. These conflicting results may reflect different experimental conditions, such as hypoxia severity, duration of exposure to hypoxia, sampling sites, and so on.

In conclusion, we confirmed that exposure to hypoxia elicits an increase in NO concentrations at relative changes of skin gas and exhaled air compared to normoxia.

## References

[1] Dweik RA, Laskowski D, Abu-soud HM, Kaneko FT (1998). Nitric oxide synthesis in the lung. J. Clin. Invest.

[2] Beall C, Laskowski D, Strohl KP, Soria R (2001). Pulmonary nitric oxide in mountain dwellers. Natur.

[3] Cremona G, Higenbottam T, Takao M, Hall L (1995). Exhaled nitric oxide in isolated pig lungs. J. Appl. Physiol.

[4] Persson MG, Wiklund NP, Gustafsson LE (1993). Endogenous nitric oxide in single exhalations and the change during exercise. Am. Rev. Respir. Dis.

[5] Gerlach H, Rossaint R, Pappert D, Knorr M (1994). Autoinhalation of nitric oxide after endogenous synthesis in nasopharynx. Lancet.

[6] Ignarro LJ (1990). Haem-dependent activation of guanylate cyclase and cyclic GMP formation by endogenous nitric oxide: a unique transduction mechanism for transcellular signaling. Pharmacol. Toxicol.

[7] Brann DW, Bhat GK, Lamar CA, Mahesh VB (1997). Gaseous transmitters and neuroendocrine regulation. Neuroendocrinology.

[8] Alving K, Weizberg E, Lundberg JM (1993). Increased amount of nitric oxide in exhaled air of asthmatics. Eur. Respir. J.

[9] Kimberly B, Nejadnik B, Giraud GD, Holden WE (1996). Nasal contribution to exhaled nitric oxide at rest and during breathholding in humans. Am. J. Respir. Crit. Care Med.

[10] Kharitonov SA, Yates D, Robbins RA, Logan-Sinclair R (1994). Increased nitric oxide in exhaled air of asthmatic patients. Lancet.

[11] Bauer JA, Wald JA, Doran S, Soda D (1994). Endogenous nitric oxide in expired air. Effects of acute exercise in human. Life Sci.

[12] Maroun MJ, Mehta S, Turcotte R, Cosio MG (1995). Effects of physical conditioning on endogenous nitric oxide output during exercise. J. Appl. Physiol.

[13] Persson MG, Zetterström O, Agrenius V, Ihre E (1994). Single-breath nitric oxide measurements in asthmatic patients and smokers. Lancet.

[14] Hample V, Cornfield DN, Cowan NJ, Archer SL (1995). Hypoxia potentiates nitric oxide synthesis and transiently increases cytosolic calcium levels in pulmonary artery endothelials cells. Eur. Respir. J.

[15] Oliveira GV, Shimoda K, Enkhbaatar P, Jodin J (2004). Skin nitric oxide and its metabolites are increased in nonburned skin after thermal injuries. Shock.

[16] Nose K, Mizuno T, Yamane N, Kondo T (2005). Identification of ammonia in gas emanated from human skin and its correlation with that in blood. Anal. Sci.

[17] Nose K, Nunome Y, Kondo T, Araki S (2005). Identification of gas emanated from human skin: methane, ethylene, and ethane. Anal. Sci.

[18] Andersen JB, Hedrick MS, Wang T (2003). Cardiovascular responses to hypoxia and anaemia in the toad Bufo marinus. J. Exp. Biol.

[19] Sheel AW, Edwards MR, Hunte GS, McKenzie DC (2001). Influence of inhaled nitric oxide on gas exchange during normoxic and hypoxic exercise inhighly trained cyclists. J. Appl. Physiol.

[20] Strijdom H, Jacobs S, Hattingh S, Page C (2006). Nitric oxide production is higher in rat cardiac microvessel endothelial cells than ventricular cardiomyocytes in baseline and hypoxic conditions: a comparative study. FASEB. J.

[21] Justice JM, Tanner MA, Myers PR (2000). Endothelial cell regulation of nitric oxide production during hypoxia in coronary microvessels and epicardial arteries. J. Cell Physiol.

[22] Arnet UA, McMillan A, Dinerman JL, Ballermann B (1996). Regulation of endothelial nitric-oxide synthase during hypoxia. J. Biol. Chem.

[23] Whorton AR, Simonds DB, Piantadosi CA (1997). Regulation of nitric oxide synthesis by oxygen in vascular endotherial cells. Am. J. Physiol.

[24] Hong Y, Suzuki S, Yatoh S, Mizutani M (2000). Effect of hypoxia on nitric oxide production and its synthase gene expression in rat smooth muscle cells. Biochem. Biophys. Res. Commun.

[25] Tsujino I, Miyamoto K, Nishimura M, Shinano H (1996). Production of nitric oxide (NO) intrathoracic airways of normal human. Am. J. Respir. Crit. Care Med.

